# Antiulcerogenic and Antibacterial Effects of Chitosan Derivatives on Experimental Gastric Ulcers in Rats

**DOI:** 10.1155/2022/4743041

**Published:** 2022-09-16

**Authors:** Leudimar Aires Pereira, Luizângela da Silva Reis, Anderson Nogueira Mendes, Hélio de Barros Fernandes, Daniel Dias Rufino Arcanjo, Adalberto Fortes Rodrigues Júnior, Janete Martins Sousa, Humberto Medeiros Barreto, Jailson de Araújo Santos, Josy Anteveli Osajima, Edson C. Silva Filho

**Affiliations:** ^1^Interdisciplinary Laboratory of Advanced Materials, Postgraduate Program in Materials Science, Federal University of Piauí, CEP: 64049-550, Teresina, Piauí, Brazil; ^2^Laboratory of Biology, Department of Biology, Universidade Federal do Piauí, 64800-000, Floriano, Piauí, Brazil; ^3^Laboratory of Innovation in Science and Technology, Department of Biophysics and Physiology, Federal University of Piauí, CEP: 64049-550, Teresina, Piauí, Brazil; ^4^Multiprofessional Residence and in Professional Health Areas, Federal University of Piauí, CEP: 64049-550, Teresina, Piauí, Brazil; ^5^Laboratory of Microbiology Research, Department of Parasitology and Microbiology, Federal University of Piauí, CEP: 64049-550, Teresina, Piauí, Brazil

## Abstract

Gastric ulcer is an injury that develops on the lining of the stomach due to an imbalance between aggressive and defensive agents. Chitosan derivatives demonstrate promising biological activities in accelerating the healing activity of gastric lesions. Thus, this study aimed at investigating the healing activity of gastric lesion, induced by acetic acid (80%), of the chitosan derivative with acetylacetone (Cac) modified with ethylenediamine (Cacen) or diethylenetriamine (Cacdien). The biological activity was determined based on cytotoxicity, antibacterial activity, and gastroprotective activities. The results showed no significant difference in the cytotoxicity, a better antibacterial activity against *S. aureus* and *E. coli*, and a positive result on the healing of gastric lesions of the materials (Cac 18.4%, Cacen 55.2%, and Cacdien 68.1%) compared to pure chitosan (50.7%). Therefore, the results indicate that derivatives of chitosan are promising biomaterials for application in the control of lesions on the gastric mucosa.

## 1. Introduction

The research and development of derivatives of natural polymers, such as chitosan, have brought promising advances for their use as biomaterials capable of promoting the wound healing process at systemic levels [1–4]. These biomaterials can activate macrophages, accelerate the process of healing, and increase the reconstruction of granulation tissue; as a result of the induction of collagen production and activation of fibroblasts, enabling the migration of polymorphonuclear neutrophils in the initial stage of the healing phase [5].

Gastric ulcer is characterized as a lesion on the lining of the stomach [6, 7]. This injury results from an imbalance between aggressive agents (secretion of gastric acid, pepsin, free radicals, psychosomatic stress, smoking, alcoholism, *Helicobacter pylori* infection, and use of nonsteroidal anti-inflammatory drugs) and defensive agents (mucus and bicarbonate secretion, nitric oxide, blood flow, sulfhydryl groups, and prostaglandins) of the digestive tract [6, 8–14]. The treatment of gastric ulcers can be compromised by serious gastric disorders against the defense of the mucosa [15]. These disorders result from serious drug side effects [13].

Chitosan is a natural cationic polysaccharide obtained from the deacetylation of chitin [16]. The chemical reaction results from substituting a part of the N-acetyl groups in chitin, which are *β*-(1-4)-2-amino-2 bonds-deoxy-D-glycopyranose and *β*-(1-4)-acetamide-2-deoxy-D-glycopyranose [17]. Chitosan shows good biocompatibility, biodegradability, permeability, low cell toxicity [3, 18], anti-inflammatory, analgesic, and hemostatic actions [19, 20]. In addition, chitosan shows antibacterial activity through an interaction between protonated amine groups with negative bacterial cell membrane and cell wall charges [5, 21], and binding as bacterial DNA, which avoids transcription and gene translation. These properties highlight the chitosan of other biomaterials in wound healing [22–24]. The antibacterial activity shown by chitosan can be improved with chemical modification in its structure, such as the incorporation of amine groups [2, 20, 25–27].

The adhesion of chitosan prevents direct contact of the injured mucosa with the physiological environment of the stomach and prevents the proliferation of microorganisms at the wound site [28, 29]. In addition, it inhibits the enzyme activities responsible for the synthesis of the microorganism's cell wall at the injured site [5].

The mucoadhesive characteristics of chitosan is attributed to the amine groups [2, 4, 18]. In an animal physiological environment, ionic bonds occur between the protonated amine groups (positive) of chitosan and the sialic acid residues (negative) of the glycoproteins present in the mucosa. This interaction causes the chitosan molecule to bind to the mucosal membrane wall and allows the bioadhesion and mucoadhesive effects [28, 29].

In its chemical structure, chitosan has two hydroxyl groups (OH) and a free amine group (NH_2_), which provide interaction with other chemical compounds such as acetylacetone [30]. Chitosan derivatives with acetylacetone show an increase in the number of active sites, which provide to its derivative a higher efficiency in the incorporation of the amine group present in compounds such as ethylenediamine or diethylene triamine [1, 25, 30]. In addition, this characteristic enhances its applications in the health area. Chitosan derivatives from acetylacetone modified with ethylenediamine or diethylenetriamine present formation of the imine bond (Schiff's base), which has antimicrobial activity [30]. This characteristic may result in the acceleration of the healing process of wounds in the gastric mucosa.

However, derivatives of natural products such as chitosan with amine groups represent promising possibilities for research and discoveries of biological activities favorable to the treatment of healing of lesions such as gastric ulcers [9]. Currently, there are no published studies on healing gastric ulcer injury by using chitosan derived with acetylacetone modified with ethylenediamine or diethylene triamine.

Thus, this study was carried out to investigate the healing activities of chitosan derivatives with acetylacetone modified with ethylenediamine or diethylenetriamine on the treatment of gastric lesion in rat induced by acetic acid (80%), as a possible application of these derivatives in the treatment or control of gastric ulcer.

## 2. Materials and Methods

### 2.1. Materials

Chitosan medium degree of deacetylation 78% (Polymar) and molar mass 132.0 KDa [25]; sodium hypochlorite, acetylacetone, ethylenediamine, and diethylenetriamine (Sigma-Aldrich, Saint Louis, USA); acetic acid (DinâmicaQuímica, Indaiatuba, SP, Brazil); brain heart infusion (BHI) (HIMEDIA, Mumbai, India), nutrient agar (HIMEDIA, Mumbai, India), GM07492A (human \fibroblast), Dulbecco's modified eagle medium-DMEM (Gibco/Thermofisher, Waltham, Massachusetts, USA), supplemented with fetal bovine serum (FBS) (Nutricell), penicillin, 10 AU·mL^−1^ streptomycin (Sigma-Aldrich, Saint Louis, USA), and sodium chloride (IMPEX); ketamine hydrochloride (10%) and 2% xylazine hydrochloride (Syntec, Barueri, SP, Brazil); sodium thiopental (Cristália, Itapira, SP, Brazil), cimetidine (TEUTO, Anápolis, GO, Brazil), and ultrapure Milli-Q® water (/Millipore Corporation, Burlington, USA) were used.

### 2.2. Chemical Modification and Characterization of Chitosan and Derivatives of Chitosan

The protocol for modifying chitosan with acetylacetone, ethylenediamine, and diethylene triamine was in accordance with Pereira et al. [25]. Chitosan and its derivatives (Cac, Cacen, and Cacdien) were characterized by elemental analysis (CHN), thermal analysis (TG/DTG/DSC), and X-ray diffractometry (DRX) according to a previous study [25] published by the research group.

### 2.3. Cytotoxicity Assessment

The cytotoxicity assay was carried out by the 3-[4, 5-dimethylthiazol-zyl]-2, 5-diphenyltetrazolium bromide (MTT) assay as previously described [31–33]. A normal human cell line (lung fibroblasts, GM07492A) were seeded in a 96-well culture plate, maintained in Dulbecco's modified eagle medium (DMEM) enriched with fetal bovine serum (FBS) and antibiotics (penicillin and 10 U/mL streptomycin) and incubated at 37°C incubator for 24 hours. The incubation step was carried out in a humidified atmosphere containing 5% CO_2_ and 95% atmospheric air. After 24 hours, the samples were weighed (10 mg) and added to 1 mL of DMEM + FBS + antibiotics. The samples were shaken for approximately 1 minute, held in ultrasound for 15 minutes, and again shaken for another 1 minute. DMEM was replaced with 100 *μ*L of the samples, which were prepared in triplicate and kept in a 37°C oven in a humidified atmosphere containing 5% CO_2_ and 95% atmospheric air, for 24 hours. Then, the wells were washed with polyphenylene sulfide (PBS), and then 50 *μ*L of MTT were added. The plate was again incubated at 37°C in an oven for 4 hours. After this period, 100 *μ*L of isopropanol was added to the wells and gently homogenized for solubilization of formazan crystals. The optical density (OD) values were obtained at 570 nm, wavelength (*λ*), in a spectrophotometer and converted into percentages of relative cell viability. As a negative control of noncytotoxic materials, it was used DMEM plus FBS and antibiotics (penicillin and 10 U/mL streptomycin (sigma)). As a positive control of cytotoxicity for the materials, DMEM plus 25% dimethyl sulfoxide (DMSO) were used.

### 2.4. Evaluation of Antibacterial Activity

The antibacterial activity assay was carried out according to Osório et al. [2]. Gram-positive strain (*Staphylococcus aureus* ATCC 25923) and Gram-negative strain (*Escherichia coli* ATCC 25922) were used in the direct contact technique. All strains were kept on nutrient agar at 4°C. In the preparation of the inoculum, cultures were obtained by transferring a range of bacterial growth on nutrient agar to a falcon tube containing 3.0 mL of brain heart infusion (BHI), followed by incubation at 37°C for 24 h. From this culture, in BHI, a bacterial suspension standardized was obtained to a density equivalent to 0.5 on the MacFarland scale (approximately 1.5 × 10^8^ CFU/mL^1^, colony forming units). Subsequently, serial dilutions were carried out in saline solution, resulting in a suspension of approximately 1.5 × 10^4^ UFC/mL and 1.5 × 10^5^ UFC/mL for *S. aureus* and *E. coli*, respectively.

The direct contact test in solid medium was carried out according to Zheng et al. [34]. In order to carry out these tests, 100 *μ*L of the standardized suspension 1.5 × 10^4^ CFU/mL was transferred to Petri dishes containing the agar nutrient medium. Then, 100 *μ*L of the test solution was added to each plate. The plates were sown with the aid of a Drigalsky loop using the spread plate method and incubated at 37°C for 24 hours. As positive control, it was used plates with nutrient agar containing the bacterial suspension and saline solution, as well as plates containing the bacterial suspension and 2% acetic acid solution. All tests were carried out in triplicate. The inhibitory antibacterial activity efficiency was calculated according to the following formula:

(1) $$\begin{array}{c} n=\frac{N_{1}-N_{2}}{N_{1}}\times 100, \end{array}$$

where *η* stands for the inhibitory effect, *N*_1_ is the arithmetic mean of the colony-forming units in the control plates, and *N*_2_ is the arithmetic mean of the colony-forming units in each tested solution. Solutions C, Cac, Cacen, and Cacdien (1000 *μ*g/mL) were used in the evaluation of antibacterial activity, which was prepared using 2% acetic acid as solvent. The tests were carried out with 0, 1, 2, 3, 4, and 5 days after the preparation of the solutions.

### 2.5. Gastric Healing Activity Test

According to F. V. da Silva et al. [12], with adaptations according to L. Servat-Medina et al. [35], the investigation of the antiulcerogenic activities of chitosan and derivatives thereof was carried out, after approval by the Ethics Committee for the Use of Animals (CEUA) from Universidade Federal do Piauí, Brazil (UFPI, approval no. 463/18), and in accordance with Brazilian Legislation for Experimental Animals regulated by federal law no. 11.794/2008.

Female Wistar rats (*n* = 6 per group; 200–320 g) were obtained from the animal facility of the Federal University of Piauí. The animals were kept under controlled conditions (24 ± 1°C), with food and free water. After 18 hours of fasting, the gastric ulcer induction protocol was carried out. Firstly, the animals were anesthetized by administering 10% ketamine hydrochloride (100 mg/kg, *b.w*.) and 2% xylazine hydrochloride (5 mg/kg, *b.w*.). Stomachs were exposed to byan2-cm abdominal incision. Thereafter, a glass cylinder with 8 mm-diameter and 2 cm-length was placed in contact with gastric serosa, wherewith 70 *μ*l of acetic acid (80%) was injected in order to induce gastric injury. The substance was keept in contact with the serosa for 1 minute, and then immediately removed. Afterwards, the injured areas were washed out with saline solution, and the stomach was inserted back into abdominal cavity, followed by suture.

After 24 hours of surgery, the animals were daily treated with vehicle solution (injured control), cimetidine (100 mg/kg; cimetidine), and chitosan and derivatives thereof (80 mg/kg) prepared in ultrapure water (pH 6.64), for seven days. On the eighth day, the animals were euthanized using sodium thiopental (150 mg/kg) and sodium lidocaine (10 mg/kg). Right after, the stomachs were removed, opened along the greater curvature, and the gastric contents were discarded. The mucosa was washed with saline solution and stretched on a Styrofoam plate in order to measure the width, height, and depth of the ulcerated area using a digital caliper. Then, the ulcer volume (cubic millimeters) was calculated as ulcerated area × ulcer depth, and values were applied to the following equation [35]:

(2) $$\begin{array}{c} \text{Gastroprotection }\left(\text{\%}\right)=\frac{\left(\text{Negative control–sample control}\right)}{\text{Negative control}}\times 100. \end{array}$$

### 2.6. Statistical Analysis

The results were shown as mean ± SEM. The statistical significance for the differences among the groups was calculated using analysis of variance (ANOVA) and Tukey's post-hoc test. Differences between the groups were considered significant when *p* < 0.05. Data were analysed and graphs were plotted using the GraphPad Prism™ 5.0 software (La Jolla, CA, USA).

## 3. Results and Discussion

Chitosan modification increased the percentage of nitrogen after each synthesis [25]. In addition, it was demonstrated that the incorporation of the amino group to the derivative Cac results in a significant increase in the percentage of nitrogen compared to pure chitosan. This study also emphasized that the chemical modification of chitosan with acetylacetone at temperatures of 123°C and the modification with ethylenediamine (140°C) or diethylene triamine at 209°C did not change the viscosity and the molecular mass of the chitosan derivatives compared to pure chitosan.

This characteristic is important since the viscosity may influence both the value of the degree of deacetylation as well as the molar mass of the polymer. Thus, the longer the chemical chain structure, the higher the molecular mass of the material. These characteristics may allow interactions between the segments and decrease the solubility of chitosan, leading to changes in the efficiency of the biological properties of these chitosan derivatives [36, 37].

**Figure 1:**
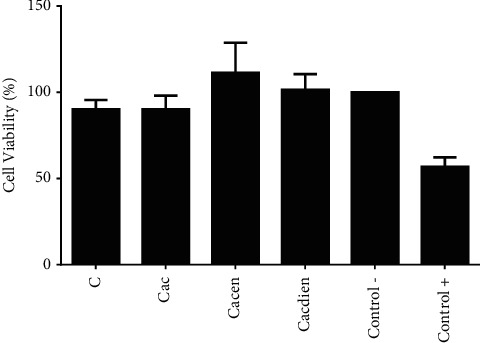
Effect of chitosan and its derivatives on the viability of GM07492A (human fibroblasts). Cell viability was assessed by the MTT assay after 24 h incubation with chitosan (C): chitosan modified with acetylacetone (Cac), chitosan modified with ethylenediamine (Cacen), and chitosan modified with diethylene triamine. Data are presented as mean ± SEM, obtained from three independent experiments (*n* = 3) in triplicate. The statistical significance for the differences among the groups was calculated using analysis of variance (ANOVA) and Tukey's post hoc test. Differences between the groups were considered significant when *p* < 0.05.

Figure 1 displays the evaluation of the cytotoxicity of different chitosans in human fibroblast strains GM07492A. According to the results of chitosan and its derivatives Cac, Cacen, and Cacdien presented low cytotoxicity, with no significant difference compared to the control. It is observed that the chemical modifications carried out in chitosan with acetylacetone and amine groups did not make the derivatives more toxic, since the cell viability obtained for the samples and no significant variations were observed compared to the positive control group (+). In the present study, the GM07492A cells (Human Fibroblast) in DMEM medium (DMEM) were used as a negative control, equivalent to 100% viability. The minimum value required for the biocompatibility test is 50% [38]. Therefore, the values obtained for toxicity to human fibroblast cells, the results of the *in vitro* hemolytic cytotoxicity test and the toxicity assays on *Artemia Salina* reported in a previous study by Pereira et al. [25] reveal that both chitosan and derivatives Cac, Cacen, and Cacdien suggest that the modified polymers may be compatible for use as biomaterials.

**Figure 2:**
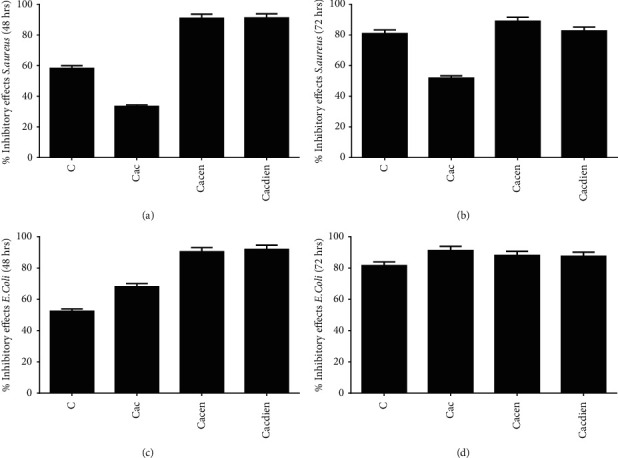
Inhibitory effect of chitosan and its derivatives on the growth of *S. aureus* and *E coli*. The inhibitory effect was evaluated after 48 h and 72 h of incubation of the bacteria with chitosan (C): acetylacetone-modified chitosan (Cac), ethylenediamine-modified chitosan (Cacen), and diethylenetriamine-modified chitosan. Data are presented as mean ± SEM, obtained from three independent experiments (*n* = 3) in triplicate.

The modified chitosan polymers were also used to investigate actions in relation to two types of bacteria: *Escherichia coli* and *S. aureus*. The results from the antibacterial activity assay of chitosan and derivatives thereof Cac, Cacen, and Cacdien are shown in Figure 2. The modification of chitosan with acetylacetone (Cac) increased the antibacterial activity of this derivative against *Escherichia coli*, but it reduced its activity against *S. aureus*.

The results presented in Table 1 indicate that the Cac derivative is the one that presents the lowest hydrophilicity. Thus, the less antibacterial activity of the Cac derivative against the Gram-positive species *S. aureus* could be related to its lower hydrophilicity due to the presence of the ketone group. This characteristic would decrease the interactions between this derivative with the anionic residues of the hydrophilic components of the wall and the Gram-positive bacteria, such as peptidoglycan and teichoic acids [39, 40].

The partition coefficient (Log *p*) indicates the hydrophobic nature of the compound. This hydrophobicity property is correlated with the biological activity of the compound [41, 42]. The quantity is considered an informative parameter of the tendency of the substance, since in the human organism, it is distributed between nonpolar structures (cell membranes) and aqueous solutions (blood plasma, lymph, and intracellular fluids).

In contrast to the results obtained with the Gram-positive strain, the Cac derivative, compared to pure chitosan, showed higher antibacterial activity against *E. coli* (Figures 2(c) and 2(d)). This higher activity may be related to the less hydrophilic of Cac, which favors higher interaction with the components of the bacterium's outer membrane. In addition, these substances with a hydrophobic character are more likely to pass through cell membranes and reach the interior of the cell at the target location where it should act [40, 43].

On the other hand, the incorporation of ethylenediamine (Cacen) and diethylenetriamine (Cacdien) improved the inhibitory effect of these two derivatives against both the species. The results of elementary analysis with the derivatives Cacen and Cacdien demonstrated that the introduction of the groups ethylenediamine and diethylenetriamine led to an increase in the amount of nitrogen in the materials compared to Cac [25]. Therefore, higher antibacterial activity of the Cacen and Cacdien derivatives could be attributed to the increase in the number of amine groups in the inserted molecules and the formation of the imine bonds formed in the derivatives. In addition, the modifications obtained in Cacen and Cacdien resulted in a higher hydrophilicity of these derivatives (Table 1), which could favor a higher interaction of these derivatives and the cell wall components from both Gram-positive and Gram-negative bacteria [8, 44, 45].

The derivatives Cacen and Cacdien showed better results of action against *E. coli*, in comparison to Cac, after 48 h and a significant result after 72 h, indicating that the action of these new materials is of a broad spectrum, being capable of inhibiting both Gram-positive as well as Gram-negative, however depending on the time of contact. This result shows that the introduction of cationic groups and imine bonds increased the ability of these materials to interact with the cell wall components of both the types of bacteria, as already verified in a previous study [2].

The Cacen and Cacdien derivatives have a higher density of positive charges due to the presence of the inserted amine groups when compared to Cac. This increase in the density of positive charge, probably, resulted in a greater interaction of the protonated amine groups present in the structure of the derivatives with the negatively charged phosphate groups present in teichoic and lipoteichoic acids in the cell wall of *S. aureus*. This result suggests that this action has caused a higher inhibition in the synthesis of new layers of peptidoglycan [46], leading to the lysis of bacteria, as suggested in a previous study for pure chitosan [2].

Although there is no specific mechanism of the action to explain the antibacterial activity of chitosan and derivatives thereof, it is possible that there was an electrostatic interaction between the molecules of chitosan and derivatives thereof positively charged with electronegative components of the cell wall and cell membrane of the tested bacteria. This interaction may have been mediated by electrostatic forces between the NH_2_ group of the Cacen and Cacdien derivatives and the negatively charged cell surface [2, 8, 47].

**Figure 3:**
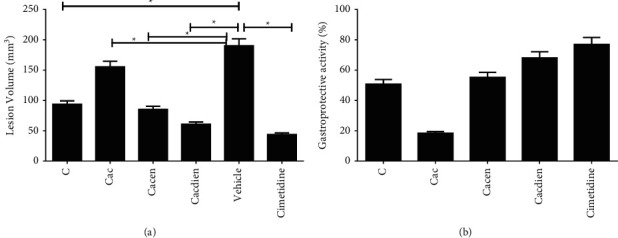
Effect of the gastroprotective activity of chitosan and its derivatives. Effect of chitosan gastroprotective activity (C): acetylacetone-modified chitosan (Cac), ethylenediamine-modified chitosan (Cacen), and diethylenetriamine-modified chitosan, cimetidine (100 mg/kg), after induction of gastric injury with acetic acid. (a) Area of residual lesion after treatment with chitosan and its derivatives. (b) Percentage of area recovered after gastric injury and treatment. Data are presented as mean ± SEM, obtained from three independent experiments (*n* = 3) in triplicate. ^*∗*^*p* > 0.5.

Table 2 shows the results of gastroprotective activity of the in vivo experiment in a rat with gastric ulcer induced by acetic acid (80%) for chitosan and its derivatives. The group of animals with ulcer without treatment showed an average of the injured area of 190.3 ± 25.4 mm^3^ (injured control/vehicle group) and the group with oral treatment with cimetidine group (daily dose of 100 mg/kg) reduced the ulcerated area by 76.9% (43.9 ± 10.2 mm^3^) compared to the injured control. Treatment with oral administration of a daily dose of 80 mg/kg for seven days, significantly, decreased the area of the ulcerative lesion by 50.7% (93.6 ± 35.5 mm^3^) for pure chitosan (Figures 3(a) and 3(b)).

These results corroborate the literature, as pretreatment with chitin and chitosan suggests an antiulcer effect, which may prevent the ulcerogenic effects induced by HCl-ethanol [48]. A lower decrease in the ulcerative lesion was presented by the derivative Cac, (18.4%/155.3 ± 21.5 mm^3^), compared to chitosan. However, this activity was enhanced with the incorporation of the amine groups (Cacen 55.2% and Cacdien 68.1%), Figure 3(b), which was more effective in regenerating the gastric mucosa compared to pure chitosan. Similar result was reported by L. Servat-Medina et al. [35] in a study with modified chitosan nanoparticles in which the dose of 30 mg/kg was sufficient to reduce 58% of gastric lesion in an experimental model of gastric ulcer induced by ethanol. Chitosan and the derivatives Cacen and Cacdien showed a statistically significant difference in the gastroprotective activity (*p* < 0.05) compared to the vehicle group.

The healing effect on the gastric mucosa damaged by acetic acid (80%), presented by the derivatives Cacen and Cacdien (Figure 3(b)), may be attributed to the antioxidant effect reported by Pereira et al. [25] for these derivatives, which corroborated to the description in the literature for derivatives of chitosan with the amine group [49]. In addition, the mucoadhesive effect of adherence to the mucosal surface presented by chitosan directly linked to the interaction of the cationic amine groups of chitosan with anionic groups on the mucosal surface. Similar effect was reported by Cardoso et al. [29] with chitosan-based hydrogels for healing skin lesions. Thus, these results can explain the lower index of healing activity presented by the Cac derivative in relation to derivatives with the amine groups (Cacen and Cacdien).

Treatments with the Cacen and Cacdien derivatives acted positively in controlling the lesion, showing a reduction of more than 55% in the ulcerated area (Figure 3(b)). This result suggests that these derivatives acted in the protection of the injured area in the gastric mucosa of the stomach. The material acts as a gastroprotective barrier and shows mucoadhesion activity as an important factor for avoiding the action of acidic elements and enzymes of the stomach [12, 15].

In addition, the direct contact with the ulcerated region with chitosan derivative allow the formation of schiff bases which has anti-inflammatory action, as previously reported by Ali et al. [47]; immunomodulatory and reparative property related to the amount of the amine group [9, 35], with efficient macrophage activation, leading to an acceleration of the healing activity of the injured region [5, 50].

**Figure 4:**
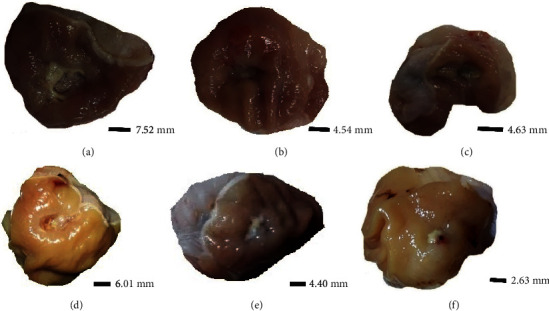
Gastric ulcer induced by acetic acid in rats treated, orally, for seven days with vehicle/injured control (a), cimetidine (100 mg/kg) (b), daily dose of 80 mg/kg of chitosan (c) and derivatives Cac (d), Cacen (e), and Cacdien (f).

Furthermore, the derivates provide an acceleration of collagen synthesis in the early stages of healing, with cytokine production, promoting the reconstitution of the injured tissue, as reported by Hajji et al. [21] in a study of wound healing activity in rats using a chitosan derivative. This characteristic justifies the healing effect close to the cimetidine control of the Cacen and Cacdien derivatives (Figure 4), suggesting that this more effective action may be attributed to the high amount of the amine group presented by these derivatives. Similar results were reported in the literature for chitosan derivatives with the amine group [5, 47, 50].

In this study, oral treatment with cimetidine, during the consecutive period for seven days, significantly, reduced gastric ulcer, which was induced by acetic acid in rats. Similarly, treatment with pure chitosan and derivatives thereof showed positive gastroprotective activities, but with a higher effect, in accelerating the healing of gastric mucosa, for derivatives with the incorporation of the amine groups.

## 4. Conclusion

Chitosan derivatives showed low cytotoxicity for human fibroblasts and compatible for use as biomaterials. The materials showed higher results of antibacterial activity against *S. aureus* and *E. coli* compared to pure chitosan. The derivatives Cacen and Cacdien demonstrated results superior to that of pure chitosan in the healing of gastric mucosa. The results suggest that these chitosan derivatives are promising agents for treatment of gastric ulcers, as well as they could also be applied as carriers of antiulcerogenic drugs, aiming obtention of an in situ release, improved gastric healing effect, and decrease of side effects.

## Figures and Tables

**Table 1:** Partition coefficient (log P) of chitosan (C) and derivatives Cacdien, Cacen, and Cac.

Log *p*	*C*	Cacdien	Cacen	Cac
	−1.51	−0.64	−0.49	−0.35

**Table 2:** Gastric healing activity induced by chitosan (C) and derivatives thereof modified with acetylacetone (Cac), ethylenediamine (Cacen), and diethylenetriamine (Cacdien) after treatment at daily doses of 80 mg/kg for seven days on acetic acid-induced gastric ulcer in rats.

Samples (groups)	Ulcer volume (mm^3^)	Gastroprotective activity (%)
Vehicle^*∗*^	190.3 ± 25.4	0.0
Chitosan	93.6 ± 35.5	50.7
Cac	155.3 ± 21.5	18.4
Cacen	85.3 ± 18.0	55.2
Cacdien	60.8 ± 28.7	68.1
Cimetidine (100 mg/kg)	43.9 ± 10.2	76.9

The results are expressed as gastric ulcer volume ± standard error of the mean. One-way ANOVA followed Tukey's post hoc test. Differences were considered significant when *p* < 0.05. ^*∗*^Injured control group (without treatment).

## Data Availability

No data were used to support the study.
